# Too Many Applicants, Too Few Training Posts: A Literature Review on the Imbalance Threatening Surgical Care in the United Kingdom

**DOI:** 10.7759/cureus.91278

**Published:** 2025-08-30

**Authors:** Abdulrahman Kashkosh

**Affiliations:** 1 Trauma and Orthopaedics Department, Doncaster and Bassetlaw Teaching Hospitals NHS Foundation Trust, Doncaster, GBR

**Keywords:** competition ratios, healthcare policy, medical education, nhs, surgical training, workforce planning

## Abstract

In the United Kingdom, surgery remains a highly popular speciality choice among medical graduates, yet prospective trainees face intense competition and limited training posts. The growing imbalance between the number of applicants and the available Core Surgical Training (CST) positions has created significant challenges for the surgical workforce pipeline. This literature review examines how increased competition and constrained training capacity create barriers for prospective trainees, impact the surgical workforce, and have downstream effects on patient care. Drawing on recent peer-reviewed studies, government reports, and workforce analyses, the current literature highlights that hyper-competition in surgical training amplifies burnout among trainees, while unfilled clinical demand exacerbates NHS waiting lists and spurs the outsourcing of surgical services. Policy experts emphasise that without expanding training posts and improving workforce planning, the United Kingdom risks a worsening shortage of surgeons, slower innovation, and compromised care quality. We conclude that strategic investments, increasing CST and Higher Speciality Training (HST) positions, supporting trainee well-being, and promoting flexible training pathways are needed to build a sustainable surgical workforce that meets population needs.

## Introduction and background

Surgery remains a highly attractive speciality among medical graduates in the United Kingdom (UK), with a London-based survey identifying it as the most popular choice among first-year students [1]. However, the pathway to becoming a consultant surgeon in the UK is lengthy, typically taking 10-12 years post graduation, and is also intensely competitive. Following medical school, trainees must complete a two-year Foundation Programme, a two-year Core Surgical Training (CST) programme, and a six-year Higher Speciality Training (HST) pathway before obtaining a Certificate of Completion of Training (CCT), often followed by additional fellowships before starting a consultant job.

Nevertheless, policy changes, including the European Working Time Directive and the 2016 junior doctor contract, have compressed training time without easing clinical demands, with the 2016 report on medical education and practice in the UK acknowledging persistent issues of disengagement and alienation among the trainees [2,3]. At the same time, while the number of UK medical graduates has risen significantly, surgical training posts have not increased proportionately. Each year, only 500-650 CST positions are available [4]. In 2020, just 605 of 2,322 applicants secured CST posts, and by 2024, competition had intensified, with over 3,300 applicants competing for 645 places [4,5].

This growing disparity between applicant numbers and available posts has created a bottleneck in surgical training, leading to heightened competition. As a result, many capable candidates are either deterred from pursuing surgery or are compelled to apply repeatedly over several years. This review examines the consequences of this rising competition and stagnant training capacity, with particular attention to its effects on trainee experience, workforce development, and patient care in the UK context.

## Review

Methods

A comprehensive narrative literature review was conducted using PubMed, Embase, Google Scholar, and official UK websites (e.g., Health Education England (HEE) (https://www.hee.nhs.uk/), Royal College of Surgeons (RCS) (https://www.rcseng.ac.uk/), NHS England (https://www.england.nhs.uk/)). Keywords included "surgical training UK," "core surgical training competition," "surgical workforce shortage," and "surgeon burnout UK." Publications from 2015 to 2024 were prioritized, but older seminal works were included when relevant. Sources included cohort studies, government reports, professional surveys, and press releases. We focused on training numbers, applicant ratios, trainee stress, workforce shortages, and patient outcomes. In-text citations correspond to the numbered reference list provided below.

Results and discussion

**Figure 1:**
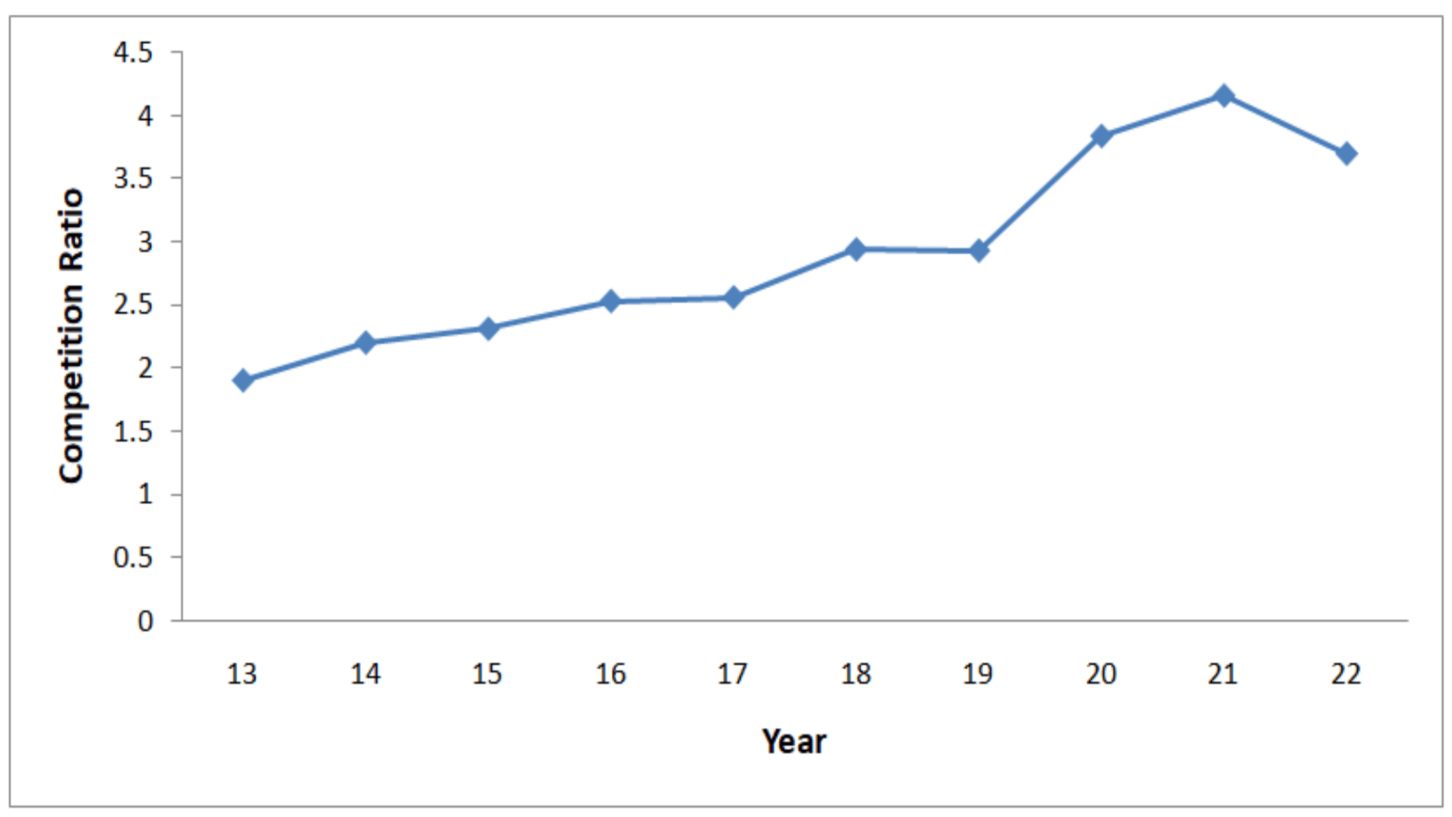
Core Surgical Training competition ratios (2013-2022) Data Source: NHS England Workforce, training and education, Competition Ratios [4] Image Credit: Abdulrahman Kashkosh

The increasing oversubscription to surgical training represents one of the most pressing challenges facing UK medical education today. CST competition ratios have escalated dramatically over the years, rising from 2.93:1 in 2019 to 4.2:1 by 2023, and reaching 5.25:1 in 2024 [4,5]. This trend is shown in Figure 1. Table 1 shows the increase in applications, reflecting the rise in UK and international medical graduates. Despite this surge, the number of available posts has remained largely unchanged, consistently averaging 620-650 annually [4]. This persistent imbalance means that even highly appointable applicants frequently fail to secure positions, creating a bottleneck that is significantly more restrictive than systems in other countries, such as the United States, where most graduates successfully match into residencies [6].

**Table 1:** Comparison of Core Surgical Training applicants and number of posts (2013-2024) Data Source: NHS England Workforce, training and education, Competition Ratios [4]

Year	Number of Applicants	Number of Posts
2024	3384	645
2023	2539	609
2022	2302	622
2021	2528	607
2020	2322	605
2019	1896	648
2018	1870	636
2017	1608	629
2016	1622	642
2015	1396	604
2014	1370	625
2013	1296	676

**Figure 2:**
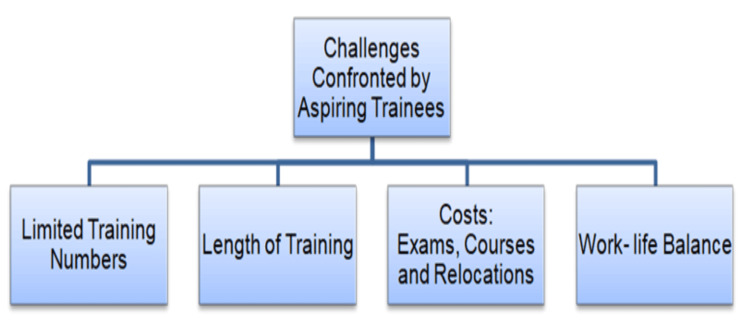
Challenges confronted by aspiring trainees Data Source: Khalil et al., 2023 [10] Image Credit: Abdulrahman Kashkosh

The intense competition has profound implications for aspiring surgeons, who consequently often undertake additional "F3" years to enhance their portfolio through operative experience, research, clinical audits, and teaching responsibilities. While these experiences may indeed provide valuable professional development, which is essential given that there is a concerning lack of surgical skills teaching that has resulted in medical students and junior doctors not having the necessary surgical skills, they nonetheless impose considerable financial and mental stress on young doctors [7]. Building upon these pressures caused by the competition, surgical training imposes additional burdens on aspiring trainees. These various challenges are summarised in Figure 2, which outlines the range of difficulties encountered by surgical trainees. The psychological impact is concerning, with research consistently demonstrating that curriculum examinations, milestone anxiety, and limited training opportunities rank among the primary stressors experienced by surgical trainees [8]. Furthermore, gender-specific challenges persist, with female trainees reporting stress related to male-dominated environments and work-family balance concerns [9]. Moreover, recent investigations examining attrition rates among surgical trainees have revealed a concerning relationship between higher competition ratios and increased dropout rates from training programs [10]. Consequently, over half of surgical trainees have considered leaving the profession, citing burnout, inadequate mentorship, and work-life imbalance [8,10]. Financially, costs include Membership of the Royal Colleges of Surgeons (MRCS) examination fees, mandatory courses, conference attendance, and travel expenses, which trainees must fund themselves [11]. Extended training duration compounds these pressures, as trainees remain in lower-paid positions longer than colleagues in other specialities. Research confirms elevated psychological distress rates among surgical trainees due to high-pressure environments, though targeted stress resilience programs show promise in reducing perceived stress and improving trainee well-being [12].

The current training bottleneck limits the number of new consultant surgeons entering the workforce, thereby constraining the NHS's capacity to meet rising surgical demand throughout the UK. This challenge is compounded by demographic trends within the existing consultant workforce, as the RCS reports that 64% of consultants aged 55-64 plan to retire within four years, with nearly 25% of the entire workforce exceeding 55 years of age [9]. This demographic shift, therefore, portends a significant shortage unless training posts undergo substantial expansion in the immediate future.

Furthermore, overstretched consultants result in limited time for mentoring, which inevitably compromises training quality for those trainees who do secure positions. The consequences extend beyond clinical training, as fewer trainees translate to reduced numbers of surgical researchers and innovators. This shortage subsequently impedes the development of novel techniques and the adoption of emerging technologies [13,14], potentially undermining the UK's position as a leader in surgical innovation. In addition to these challenges, the COVID-19 pandemic has further exacerbated these difficulties, with quantitative studies demonstrating significant reductions in surgical training opportunities across all specialities during 2020 [15]. Analysis of specific surgical subspecialties, such as vascular surgery, has revealed predictions of critical consultant shortages that could severely compromise patient care delivery [16].

These workforce challenges have direct and concerning implications for patient care delivery. With fewer surgeons entering the profession, existing consultants face increasing workloads and case complexity, creating a concerning cycle of overwork and burnout. This situation, alongside the COVID-19 pandemic, has consequently contributed to NHS waiting lists reaching over 7.5 million patients in England [17], representing an unprecedented crisis in healthcare service provision. In response to these capacity constraints, there has been increased reliance on outsourcing surgical procedures to private providers. Nevertheless, research indicates that such outsourcing correlates with higher treatable mortality rates, potentially attributable to variations in care quality and continuity [18]. Additionally, unsafe working conditions further compromise surgical outcomes and patient safety [13], thus establishing a detrimental feedback loop that affects both healthcare professionals and the patients under their care. A comprehensive analysis of postgraduate surgical training by Khalil et al. has highlighted the disconnect between trainees' perspectives and institutional approaches to addressing these systemic issues [10].

While the UK confronts these significant challenges, it is nevertheless essential to contextualise these issues within an international framework. Other nations face comparable workforce pressures, though their healthcare systems often employ different approaches to address them. For instance, in the United States, a greater proportion of medical graduates successfully enter residency programs due to more comprehensive funding mechanisms and local graduate prioritisation [6]. Nevertheless, the Lancet Commission on Global Surgery recommends a minimum standard of at least 20 surgical providers per 100,000 population [19]. Although the UK currently meets this threshold, its ageing population and increasing disease burden necessitate continued growth in surgical capacity to maintain adequate care standards.

## Conclusions

The persistent imbalance between rising applicant numbers and stagnant surgical training posts in the UK creates systemic challenges that reverberate throughout the healthcare system. Trainees experience increased stress and burnout, whilst consultant shortages strain healthcare delivery at multiple levels. Most critically, patient care suffers through prolonged waiting times and reduced access to safe surgical services.

To address these interconnected issues, the UK must therefore implement decisive action across multiple domains. Specifically, the healthcare system must expand CST and HST posts to align with demand, invest substantially in training infrastructure and mentorship programs, provide comprehensive financial and well-being support to trainees, and establish robust planning mechanisms for future retirements and evolving service requirements. Ultimately, strategic workforce planning and sustained investment are essential to ensure the NHS maintains high-quality surgical care and meets the needs of a growing and ageing population. Without these interventions, the current crisis will undoubtedly continue to intensify, with far-reaching consequences for both healthcare professionals and the patients who depend on their expertise.
